# Ablative Radiotherapy Reprograms the Tumor Microenvironment of a Pancreatic Tumor in Favoring the Immune Checkpoint Blockade Therapy

**DOI:** 10.3390/ijms22042091

**Published:** 2021-02-19

**Authors:** Yu-Hung Lee, Ching-Fang Yu, Ying-Chieh Yang, Ji-Hong Hong, Chi-Shiun Chiang

**Affiliations:** 1 Department of Biomedical Engineering and Environmental Sciences, National Tsing Hua University, Hsinchu 30013, Taiwan; s102012018@yahoo.com.tw; 2 Radiation Biology Research Center, Institute for Radiologic Research, Chang Gung University/Chang Gung Memorial Hospital, Taoyuan 333323, Taiwan; chingfang@mail.cgu.edu.tw (C.-F.Y.); jihong@cgmh.org.tw (J.-H.H.); 3 Radiation Oncology, National Taiwan University Hospital Hsin-Chu Branch, Hsinchu City 300195, Taiwan; ycyangtobe@gmail.com; 4 Department of Radiation Oncology, Chang Gung Memorial Hospital Linkou Branch, Taoyuan 333423, Taiwan; 5 Department of Medical Imaging and Radiological Sciences, Chang Gung University, Taoyuan 333323, Taiwan; 6 Institute of Nuclear Engineering and Science, National Tsing Hua University, Hsinchu 30013 Taiwan; 7 Frontier Research Center on Fundamental and Applied Sciences of Matters, National Tsing Hua University, Hsinchu 30013, Taiwan

**Keywords:** pancreatic cancer, radiation therapy, PD-L1, PD-1, CD8+ T cells, tumor microenvironment

## Abstract

The low overall survival rate of patients with pancreatic cancer has driven research to seek a new therapeutic protocol. Radiotherapy (RT) is frequently an option in the neoadjuvant or palliative settings for pancreatic cancer treatment. This study explored the effect of RT protocols on the tumor microenvironment (TME) and their consequent impact on anti-programmed cell death ligand-1 (PD-L1) therapy. Using a murine orthotopic pancreatic tumor model, UN-KC-6141, RT-disturbed TME was examined by immunohistochemical staining. The results showed that ablative RT is more effective than fractionated RT at recruiting T cells. On the other hand, fractionated RT induces more myeloid-derived suppressor cell infiltration than ablative RT. The RT-disturbed TME presents a higher perfusion rate per vessel. The increase in vessel perfusion is associated with a higher amount of anti-PD-L1 antibody being delivered to the tumor. Animal survival is increased by anti-PD-L1 therapy after ablative RT, with 67% of treated animals surviving more than 30 days after tumor inoculation compared to a median survival time of 16.5 days for the control group. Splenocytes isolated from surviving animals were specifically cytotoxic for UN-KC-6141 cells. We conclude that the ablative RT-induced TME is more suited than conventional RT-induced TME to combination therapy with immune checkpoint blockade.

## 1. Introduction

Pancreatic ductal adenocarcinoma (PDAC) is one of the most challenging cancers. According to SEER (Surveillance, Epidemiology, and End Result), the 5-year overall survival (5YOS) rate of many cancers has been significantly improved [1], but the 5YOS for PDAC is less than 10%, which is the lowest of all cancers and has not improved in the last ten years [2]. Currently, surgical resection seems the only possibility for cure [3], but most patients have locally advanced tumors involving a regional critical vascular structure or distant metastatic spread [4,5,6]. Unresectable patients typically receive neoadjuvant chemotherapy or chemo-radiotherapy to shrink the tumors to a resectable size before surgery [7,8]. Despite the improvement in surgery, patient care, and first-line and second-line therapy, the PDAC still has a dismal prognosis with a recurrence rate as high as 80%.

Recent progress in cancer immunotherapy (CIMT) holds great promise for increasing cancer cure rates, as is shown in melanoma by immune checkpoint blockade therapy (ICBT) [9,10]. Targeting programmed cell death protein-1 (PD-1), programmed cell death ligand-1 (PD-L1), and cytotoxic T lymphocyte-associated protein-4 (CTLA-4) therapy has resulted in responses in various tumors such as Hodgkin’s lymphoma and non-small cell lung cancer [11,12]. However, ICBT has not yet been successfully translated into PDAC [13], where clinical trials have shown poor responses to mono-ICBT [14,15]. PDAC frequently develops a unique tumor microenvironment (TME) composed of the high density of stromal cells and multiple immunosuppressive mechanisms [16,17,18]. Due to these multiple obstacles, an immune boost to overcome the immunosuppressive pancreatic TME is critically needed. In this regard, encouraging results have been reported in recent clinical trials (NCT02588443) of a combination of anti-CD40 with Nab-paclitaxel and gemcitabine to target different stromal components.

Radiotherapy (RT) is an immune modulator [19,20,21,22]. Clinical cases showed the addition of RT to anti-CTLA-4 treatment significantly prolonged the survival of advanced melanoma. A rare abscopal effect was induced with immune regression of untreated tumors at a distant site from that of irradiation [23,24] and it seems that local RT can increase the presentation of a neo-antigen within the tumor stroma, thereby provoking anti-tumor immunity [25]. The immune-modulating effect of RT resulted in increased CD8+ T cell infiltration, release of neo-antigens, or relieving immune suppression. However, the immune response that is generated varies with the RT schedule and tumor model. For example, single ablative dose radiation could result in decreased levels of intra-tumoral myeloid-derived suppressor cells (MDSCs) via TNF-α overexpression in a murine colon cancer model [26]. On the other hand, an increase of circulating MDSCs following conventional fractionated RT was reported in murine prostate cancer [27,28].

RT is only rarely used alone in treating pancreatic cancer patients because of the spatial location of this type of tumor nearby to the GI system. The high sensitivity of the GI limits the radiation dose in treating PDAC patients. However, advances in stereotactic body irradiation therapy (SBRT), proton therapy, or heavy ion therapy can minimize the radiation dose to the critical organ, enhancing the number of PDAC patients that can be treated with RT [29,30]. This study aimed to compare the effect of an ablative RT protocol versus a fractionated one on the TME of pancreatic tumors and its subsequent impact on the combination with anti-PD-L1 ICBT. This study illustrated that the TME resulting from ablative RT was more suited for supporting ICBT. The combination of RT and anti-PD-L1 therapy prolonged the survival of tumor-bearing mice by suppressing local tumor growth and metastasis.

## 2. Results

### 2.1. The Effect of Radiotherapy on Pancreatic Tumor

**Figure 1:**
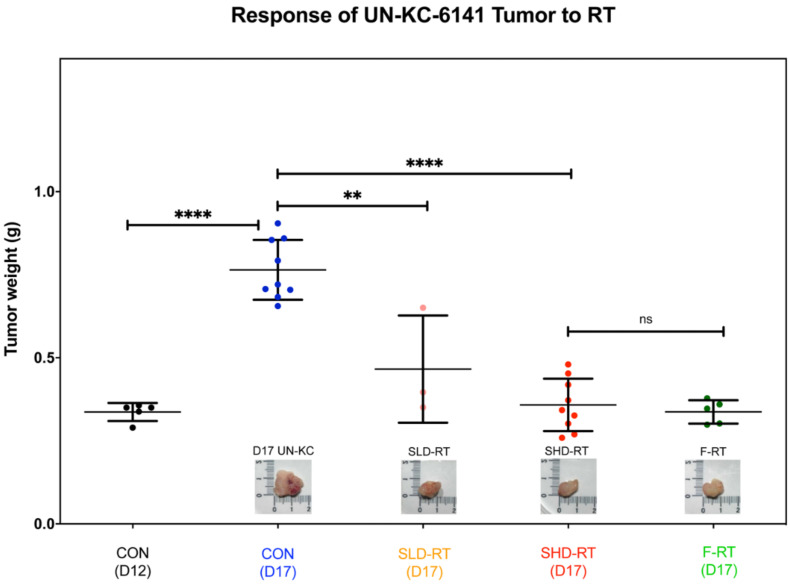
Radiotherapy (RT) inhibited the growth of pancreatic tumor UN-KC-6141 effectively. Mice bearing orthotopic UN-KC-6141 pancreatic tumors were irradiated on day 12 by various RT protocols, a single dose of 10 Gy (SLD-RT), a single dose of 25 Gy (SHD-RT), and four times 10 Gy per fraction (F-RT). Mice were euthanized on day 17, and the tumor was weighed. The photos are the representative results of tumors in each group. N ≥ 3 per group. **: *p* < 0.01; ****: *p* < 0.001.

To examine the effect of RT on pancreatic tumors, a murine orthotopic UN-KC-6141 tumor model was used [31]. Tumor-bearing mice were irradiated on day 12 after inoculating tumor cells orthotopically into the pancreas. The RT protocols examined in this study were a single dose of 10 Gy (SLD-RT), a single dose of 25 Gy (SHD-RT), and four times 10 Gy per fraction (F-RT). In the linear-quadratic cell survival model, the α/β ratio is the dose where α parameter and β parameter make the same contribution to radiation killing and this model can be used to estimate the effect of changes in dose per fraction on total dose. Early responding tissues including many tumors generally have a higher α/β value than late responding tissues such as the spinal cord or kidney. Based on this model, the F-RT protocol has a similar biological effective dose (BED) as the 25 Gy of SHD-RT, assuming α/β = 10. The effect of RT was determined by the tumor weight at day 17 (five days after irradiation). The results showed that the ability of RT to cause tumor-shrinkage was dose-dependent, and the SHD-RT protocol had a similar effect on tumor shrinkage as the F-RT protocol with the same BED (Figure 1).

### 2.2. The Effect of Ablative Radiotherapy (RT) on The Immune Microenvironment of Murine Pancreatic Tumor

**Figure 2:**
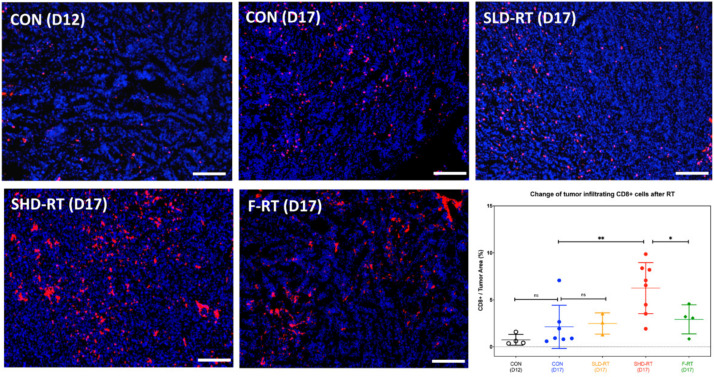
The number of tumor-infiltrating CD8^+^ cells had a significant increase after SHD-RT. The representative figures for CD8^+^ (red) staining of tumor tissues from different experimental groups are shown, and the percentage of CD8^+^ cells was quantified. The cell nucleus was stained by 4′,6-diamidino-2-phenylindole (DAPI, blue). Open circle: day12 control group; blue filled circle: day17 control group; yellow filled triangle: day 17 SLD-RT group; red filled circle: day17 SHD-RT; green filled diamond: day17 F-RT. Each dot represents one mouse data, and each mouse data contain five tumor sections counted. Scale bar: 200 μm. NS: not significant difference; *: *p* < 0.05; **: *p* < 0.01.

RT is a well-known immune modulator that can provide danger signals to awaken the immune system [19,21,22]. Numerous preclinical cancer models including our research in brain and prostate tumor models have shown that RT can change the TME [22,28,32,33,34,35,36,37] and provoke anti-tumor immune responses locally and systemically [26,28,38,39,40]. To examine the effect of RT on the immune microenvironment of UN-KC-6141 tumors, the expression of CD8 and CD4 in control, SHD-RT-, or F-RT-treated tumors was examined by immunohistochemical (IHC) staining. The IHC imaging showed that the irradiated tumor had increased CD8+ (Figure 2) and CD4^+^ (Figure S1) cells. However, only CD8^+^ cells were significantly higher in SHD-RT-treated tumors than in other groups (Figure 2), even though the SHD-RT- and F-RT-treated tumors were of similar tumor mass (Figure 1). The results illustrated that SHD-RT treatment had a more substantial effect than F-RT on recruiting CD8^+^ T lymphocytes. Moreover, the percentage of PD-1^+^ among CD8+ cells increased after SHD-RT (Figure S2A,B).

To further examine whether the increased CD8^+^ cells contributed to the killing effect of SHD-RT, tumor-bearing mice were injected intraperitoneally (i.p.) with three doses of anti-CD8 monoclonal antibody (10 mg/kg) immediately after SHD-RT (Figure S2C). The depletion efficacy was confirmed by measuring the circulating and intra-tumoral CD8^+^ levels. The data showed that this protocol could effectively deplete circulating and tumor-infiltrating CD8^+^ T cells (Figure S2D). However, it did not affect the reduction in tumor weight by RT (SHD-RT: 0.358 ± 0.079 g vs. SHD-RT + αCD8: 0.445 ± 0.007 g, *p* = 0.463), which might indicate that the CD8+ cells in irradiated tumors might be functionally suppressed.

**Figure 3:**
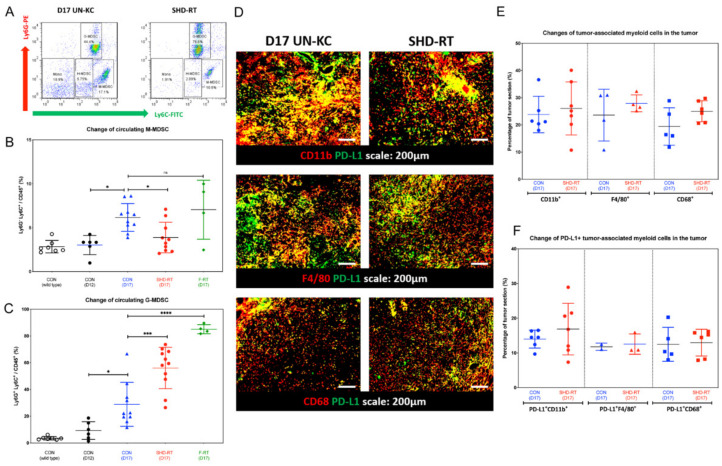
SHD-RT induced more myeloid-derived suppressor cells (MDSCs) in peripheral blood. (**A**) The gating strategy is shown for analyzing myeloid cells in the blood. Four groups of CD11b^+^ myeloid cells were analyzed: Ly6G^+^Ly6C^+^ G-MDSC, Ly6G^-^Ly6C^hi^ M-MDSC, Ly6G^-^Ly6C^lo^ H-MDSC, and Ly6G^-^Ly6C^-^ monocytes. The percentage of (**B**) M-MDSC and (**C**) G-MDSC was quantified. *: *p* < 0.05; ***: *p* < 0.005; ****: *p* < 0.001. Open circle: normal mice; filled circle: day12 control group; filled blue triangle: day17 control group; red filled circle: day 17 SHD-RT group; green filled diamond: day17 F-RT group. (**D**) The representative IHC figures of the expression of tumor-associated myeloid cell marker, CD11b, F4/80, CD68, and PD-L1. (**E**,**F**) The percentage of CD11b^+^, F4/80^+^, CD68^+^ tumor-associated myeloid cells, and PD-L1^+^ tumor-associated myeloid cells in the tumor sections were quantified. Blue filled circle, filled triangle and filled square: day17 control group; red filled circle, filled triangle and filled square: day17 SHD-RT group.

To further explore the immune profile in tumor-bearing mice following RT, peripheral myeloid cells were analyzed by flow cytometry using CD45, CD11b, Ly6G, and Ly6C antibodies. Using our gating strategy (Figure 3A), circulating CD11b^+^ myeloid cells in peripheral blood could be classified into four subpopulations: CD11b^+^Ly6G^+^Ly6C^+^ granulocytic-MDSCs (G-MDSCs), CD11b^+^Ly6G^–^Ly6C^hi^ monocytic-MDSCs (M-MDSCs), CD11b^+^Ly6G^–^Ly6C^lo^ heterogeneous myeloid-derived cells (H-MDSCs), and CD11b^+^Ly6G^–^Ly6C^–^ monocytes. Compared with age-matched control mice, tumor-bearing mice had increased levels of M-MDSCs and G-MDSCs in the blood as the disease progressed (Figure 3B,C, respectively). Notably, SHD-RT, but not by F-RT, significantly decreased the M-MDSCs levels (Figure 3B). On the other hand, both SHD-RT and F-RT increased the proportion of G-MDSCs (Figure 3C). Tumor-associated myeloid cells within the tumor were also examined by IHC. The results showed that the proportions of tumor-associated myeloid cells, regardless of staining by CD11b, F4/80, or CD68, were not significantly changed in SHD-RT-treated tumors (Figure 3D,E). We also noted that around 50% of these cells expressed PD-L1, which was not significantly altered by SHD-RT-treatment (Figure 3F). These results indicated that the TME in pancreatic tumors was strongly immune suppressive, and this was not relieved after SHD-RT, despite an increase in infiltrating CD8+ T cells.

### 2.3. The Effect of Ablative RT on Tumor Vessel Network

**Figure 4:**
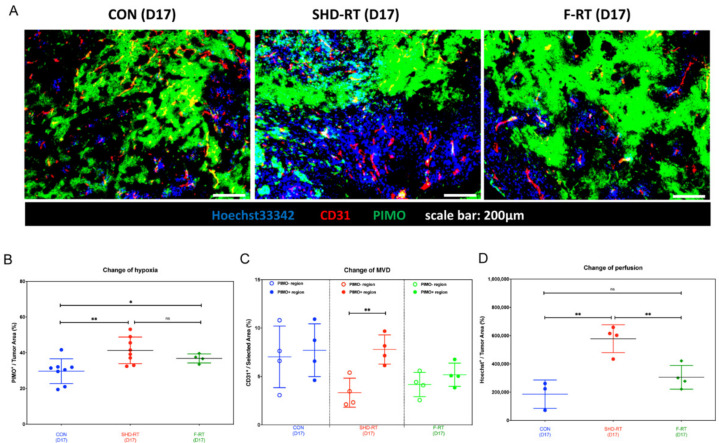
SHD-RT increased the perfusion ability of tumor vessels. (**A**) Representative figures of CD31^+^ vessels (red), PIMO^+^ hypoxia region (green), and Hoechst33342^+^ (blue) in day 17 tumors of control, SHD-RT and F-RT. (**B**) The percentage of PIMO^+^ hypoxia region was quantified in each tumor. (**C**) The MVD of PIMO^+^ and PIMO^-^ regions was quantified, respectively. (**D**) The perfusion ability of vessels was measured by the Hoechst33342^+^ area in tumors. *: *p* < 0.05; **: *p* < 0.01.

In addition to tumor-associated stromal cells, the vascular network is a crucial TME factor affected by RT [41,42]. To investigate the effects of RT on the vascular network of the UN-KC-6141 tumor, the endothelial marker, CD31, and hypoxia marker, PIMO, were examined by IHC. Comparing the tumor sections of the control mice, RT significantly decreased the microvasculature density (MVD) (Figure 4A), which was associated with an increase in tumor hypoxia (Figure 4B). The tumor section could be further divided into PIMO positive and PIMO negative regions. The results showed that SHD-RT mainly decreased the vessels within the PIMO negative regions, and F-RT had similar degrees of damage to PIMO negative and positive areas (Figure 4C). It appears that SHD-RT and F-RT had a different effect on tumor vessel networks. The staining area of the Hoechst33342 dye was further used to evaluate the vessel perfusion ability. The results showed that only SHD-RT, but not F-RT, could significantly increase the perfusion area compared with the control tumor (Figure 4D). To explore the reasons for the better vessel perfusion in SHD-RT-treated tumors, the pericyte marker, NG2 (Figure S3A), was also stained to examine the vessels’ integrity. The IHC data showed that the vessels in SHD-RT-treated tumors had lower pericyte coverage after SHD-RT (Figure S3B). This result suggested that the better perfusion in SHD-RT-treated tumors might result from increased leakage of immature vessels rather than vascular normalization. The data also indicate that the surviving vessels after SHD-RT might favor the penetration of small molecule drugs.

### 2.4. The Effect of Ablative RT with Immune Checkpoint Blockade Therapy for Pancreatic Tumor

**Figure 5:**
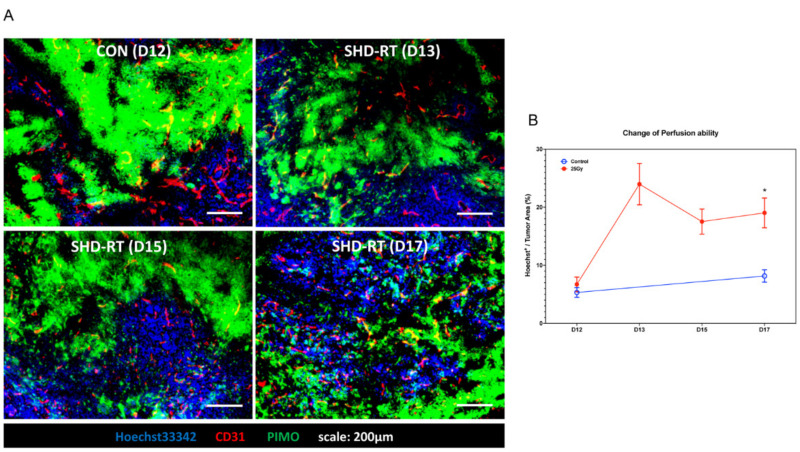
SHD-RT enhanced the perfusion ability of vessels. (**A**) Representative figures of CD31^+^ vessels (red), hypoxia (green), and Hoechst33342 (blue) in tumors of SHD-RT on a different day. (**B**) The perfusion ability of vessels was measured by the Hoechst33342^+^ area in tumors. *: *p* < 0.05.

The above results indicated that the TME in SHD-RT-treated tumors had higher perfusion ability that might favor adjuvant drug delivery. The timing of enhanced perfusion could be critical to adjuvant therapy, as was proposed for the vessel normalization model [43]. Therefore, we examined whether there was a particular window of SHD-RT-enhanced perfusion by analyzing the tumor samples at various times after SHD-RT. The results (Figure 5A) showed the changes in MVD, hypoxic area, and perfusion ability of the control (day12) and SHD-RT (day13, day15, and day17) groups. This study demonstrated a significant increase in MVD in the control tumor with time, but the MVD within the SHD-RT-treated tumor was decreased one day after RT and remained low up to five days after RT. On the other hand, the perfusion area was increased one day after F-RT and remained high up to five days (Figure 5B). These results indicated that at least five days after the SHD-RT was a convenient window for administering any adjuvant.

**Figure 6:**
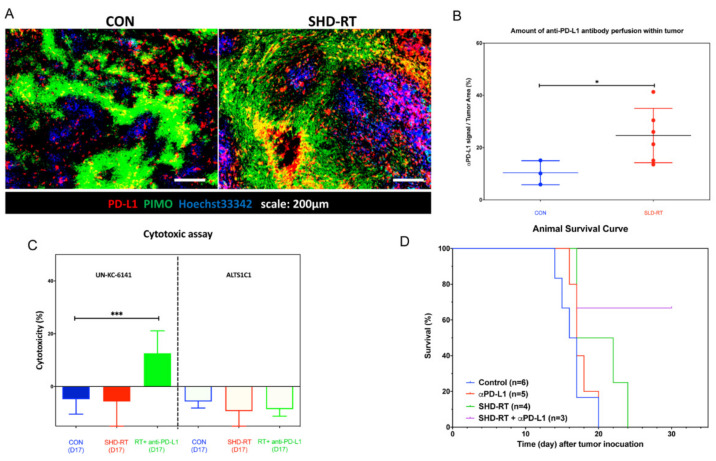
The combination of SHD-RT and anti-PD-L1 antibody prolonged the survival of tumor-bearing mice. (**A**) Representative figures of PD-L1 (red), hypoxia (green), and Hoechst33342 (blue) in day 17 tumors of SHD-RT. (**B**) The amount of anti-PD-L1 antibody perfused with tumors was determined. *: *p* < 0.05. (**C**) The specific cytotoxic activity of the splenocytes isolated from each group was examined by co-cultured with parental cells, UN-KC-6141, and murine astrocytoma cells, ALTS1C1. ***: *p* < 0.001. (**D**) Kaplan–Meier survival curves of control, PD-L1, SHD-RT, and SHD-RT + PD-L1 groups.

The above result (Figure 3) showed that the TME after SHD-RT was still highly immune-suppressive despite increased PD-1^+^CD8^+^ T cell infiltration (Supplementary Figure S2B). To explore the potential of anti-PD-L1 adjuvant therapy following SHD-RT, an anti-PD-L1 antibody was administered by i.p. injection on the following five days. The results showed that the tumor weight of the combination group (0.257 ± 0.039 g) was significantly less than that of the SHD-RT only (0.358 ± 0.079 g, *p* < 0.05) or mono-anti-PD-L1 group (0.867 ± 0.187 g, *p* < 0.05). Since SHD-RT improved whole tumor perfusion (Figure 4D and Figure 5C), whether the enhanced efficacy in the combination group resulted from increased drug delivery was examined. Using anti-PD-L1 IHC staining (Figure 6A), more anti-PD-L1 accumulated in SHD-RT-treated tumor tissues (Figure 6B), which supported the view that the increased perfusion did favor drug delivery.

To examine whether combination therapy could relieve immune suppression and stimulate a specific immune response, a cytotoxic T lymphocyte (CTL) assay of splenocytes was performed against UN-KC-6141 or ALTS1C1 (murine astrocytoma) targets [44]. Results showed that the splenocytes from the combined group had significant cytotoxicity to UN-KC-6141 cells, but not ALTS1C1 cells (Figure 6C). The data indicated that the increased perfusion and delivery of anti-PD-L1 antibody as a result of SHD-RT could overcome immune suppression. 

Most of the abdominal organs such as the intestine and liver were exposed inevitably when a large RT field (36 cm × 1 cm) was used to irradiate a group of mice simultaneously. In general, the RT causes a side effect of intestinal damage that can become lethal 5–7 days post-irradiation, which may limit the observation time after the therapy. To examine whether a smaller irradiation field (1 cm × 1 cm) could extend the observation window, the same combination schedule was used. The results showed that the survival time for mice receiving combined treatment with a small SHD-RT field was significantly improved (Figure 6D) to 24 days (12 days after RT) compared with the other groups (control: 16.5 days, αPD-L1: 17 days, SHD-RT: 19.5 days). Two of three mice survived more than 30 days, despite the fact that they were not tumor-free. At the end of the assay, secondary tumors were always found in the abdominal cavity of the control mice, and in mice receiving SHD-RT treatment and combined anti-PD-L1 therapy and sacrificed at day 17 or day 30, respectively, no secondary peritoneal tumors emerged in the combined group, but always in the control, anti-PD-L1, or SHD-RT single treatment groups. These results indicated that the development of a specific anti-UN-KC-6141 immune response in combined therapy not only inhibited the local tumor growth, but also had an abscopal effect on secondary tumor formation.

## 3. Discussion

Due to the adverse toxicity to abdominal organs, conventional RT is rarely used in treating pancreatic cancer patients. However, with the advances in RT technology such as SBRT, the high dose of radiation can be precisely delivered to target tissues while sparing the radiation dose to normal tissues. This study demonstrated that a high dose of RT could alter the TME of the UN-KC-6141 tumor to favor T cell infiltration and the delivery of anti-PD-L1 antibody. The results also illustrated the potential of a small field of high dose ionizing radiation combining with an immune checkpoint blockade agent, anti-PD-L1 antibody, for treating PDAC in an orthotopic murine pancreatic tumor model.

As an immune modulator, SHD-RT could result in a higher amount of T cell infiltration than F-RT in the UN-KC-6141 model even though the tumor microenvironment was highly immunosuppressive. The radiation-induced T cell infiltration increase is an essential element for the abscopal effect to eradicate the distant metastasis. The finding of irradiation-elicited T cell infiltration was similar to previous reports in the B16 melanoma tumor model, which had shown that the ablative radiation (20 Gy) was more effective at inducing CD8^+^ T cell infiltration and reducing metastasis than fractionated radiation or chemotherapy [45]. However, in some tumor models such as murine adenocarcinoma TSA breast cancer, the fractionated protocol was more effective than the single-dose protocol at inducing the abscopal effect when combined with anti–CTLA-4 therapy [38]. The different outcomes may be associated with the type of tumor and their associated TME. In general, the TME can be divided into two types. Inflamed tumors such as melanoma have a higher content of infiltrating T cells and frequently favor ICBT. The other is the uninflamed tumor, which is, in general, a non-immunogenic tumor such as the CT26 colon cancer, which have low CD8^+^ T cell infiltration and less response to vaccination [46]. When CT26 was given a single dose of 30 Gy, 13 of 15 mice showed complete remission with robust CD8^+^ T cell infiltration; however, fractionated (10 × 3 Gy/fraction) was ineffective [26]. It has been reported that F-RT might harm effector cells in some situations [47]. For example, the 30 Gy treatment’s therapeutic effect was nullified by additional fractionation [26], which might be related to the radio-sensitivity of T cells, lymphoid organs, and bone marrow cells being relatively more sensitive to irradiation than other cells [48]. The extended radiation time in F-RT protocol might injure those effectors cells during their recruitment journey from the bone marrow or lymphoid organs.

The UN-KC-6141 model used in this study, similar to the CT26 tumor model [26], could be considered as an “uninflamed” or “cold” tumor with relatively low numbers of CD4^+^ (~1%) and CD8^+^ (~2%) T cells and no response to anti-PD-L1 therapy because only 12.5% CD8^+^ T cells express PD-1. This study demonstrated that a high dose of ablative radiation therapy successfully turned a cold tumor into one responsive to anti-PD-L1 immunotherapy. In addition to the increase of infiltrating PD-1 positive CD8 T cells, perfusion might be one of the critical elements for improving the effect of combination therapy. Since the concept of enhanced permeability and retention (EPR) for drug delivery failed in the clinic [49,50], how to effectively use unique TME features to enhance the efficacy of cancer therapy has become an important research issue. This study demonstrated that SHD-RT effectively enhanced tumor perfusion (Figure 4D) and improved the effective delivery of anti-PD-L1 antibody (Figure 6B) in this preclinical pancreatic tumor model. These indicated that the surviving vessels after SHD-RT in the pancreatic tumor favored antibody penetration.

The RT-improved drug perfusion window reported in this study may differ from the so-called “vessel normalization” window proposed by Jain [51]. They illustrated that the immature structure and poor function of tumor vessels reverted to mature structures with better permeability after treatment with anti-angiogenesis drugs. In this study, the remaining vessels in RT-treated UN-KC-6141 tumors were more similar to the control immature tumor vessels because they had lower NG2 coverage (Figure S3). The RT-increased perfusion could be the consequence of RT-induced inflammatory vasorelaxation [52,53], decreased interstitial fluid pressure [54], or improved tumor circulation [55].

In conclusion, this study found a five-day’ radiation-enhanced perfusion window immediately after the RT for following ICBT. The low survival rate of PDAC patients is mainly due to the spread of tumor cells to other organs in most patients. The CIMT could be the best strategy to target those metastasized cancers, but CIMT alone has not successfully treated PDAC patients. This study demonstrates that a small field of RT plus ICBT improved overall survival time and diminished the development of secondary tumors. The splenocytes from these groups of mice also developed specific cytotoxicity against the parental tumor, indicating the induction of long-term immunity. Our results show the limitation of conventional RT for PDAC treatment. On the other hand, the results present the potential of precision RT for treating PDAC. The greater prolonged animal survival resulting from small field RT protocol provided preliminary data to support advanced SBRT or particle therapy combined with an immune checkpoint inhibitor for an improved local and systemic anti-tumor response against advanced PDAC.

## 4. Materials and Methods

### 4.1. Cell Line Culture

The murine pancreatic ductal adenocarcinoma cell line, UN-KC-6141, was a gift from Prof. Batra, University of Nebraska Medical Center, USA [31]. UN-KC-6141 cells were incubated at 37 °C/5% CO_2_ humidified air condition and maintained in Dulbecco’s modified Eagle’s medium (Gibco, Grand Island, NY, USA) with 10% fetal bovine serum (Gibco) and 1% penicillin-streptomycin (Gibco). Lack of Mycoplasma contamination was confirmed by the EZ-PCR™ Mycoplasma Detection Kit (Biological Industries, Beit Haemek, Israel) before the experiments.

### 4.2. Orthotopic Implantation of UN-KC-6141 Cells

C57BL/6J mice aged 8–10 weeks old were purchased from the National Laboratory Animal Center of Taiwan. All experiments and animal handling were conducted according to the guidelines under the approval of the Institutional Animal Care and Use Committee of National Tsing Hua University, Taiwan (IACUC No.: 10419). The procedures of implantation were briefly described. The cells were harvested and washed twice in a serum-free medium to remove the excess proteins. The cell suspension was mixed with Matrix Gel (Basement Membrane Growth Factor Reduced Phenol-Red Free, CORNING, Corning, NY, USA) at a ratio of 1:1 and kept on ice. Mice were anesthetized by i.p injection of ketamine (50 mg/kg, Merial Laboratoire de Toulouse, France) and xylazine (20 mg/kg, Bayer HealthCare Animal Health, Germany). The pancreas was surgically exposed through an abdominal excision. Tumor cells (1 × 10^5^ cells) in a volume of 20 μL were injected into the subscapular region of the pancreas with a 27-gauge needle. A cotton swab was used to prevent leakage after injection, and the wound was closed and covered with ointment to prevent the infection.

### 4.3. Radiation Therapy Procedures

To examine the efficacy of RT, at day12 after orthotopic implantation, mice were randomized into four groups including the control, a single low dose of 10 Gy radiation therapy (SLD-RT), a single high dose of 25 Gy radiation therapy (SHD-RT), and fractionated radiation therapy (F-RT) with 10 Gy per day for four days. Mice were anesthetized by i.p injection of ketamine and xylazine and restrained by adhesive tape during irradiation. Mice were irradiated by 6-MV x-rays from a linear accelerator of Taiwan University Hospital, Hsinchu branch, and Chang Gung Memorial Hospital with a dose rate of 2 to 3 Gy/min and covered a 1.5-cm bolus on the surface. The xiphoid process was the marker of the body surface for alignment. The irradiation field was started from 0.4 mm below the low edge of the xiphoid process with a width of 1 cm. Two kinds of RT fields were examined in this study. The irradiation setup was performed in a large field of 36 cm × 1 cm (length × width) to irradiate a group of mice simultaneously. The other was the small RT field (1 cm × 1 cm) to target the pancreatic region of one mouse per irradiation. The efficacy of treatment was determined by tumor weight at five days after RT. To investigate the response of the tumor microenvironment to different RT schedules, the tumor was analyzed by immunohistochemical staining and flow cytometry. The blood was harvested to detect the circulating MDSC populations by flow cytometry.

### 4.4. Immunotherapy Procedures

To examine the efficacy of immunotherapy, mice were randomized into the following group, control, SHD-RT, αPD-L1, and combination group. The in vivo PD-L1 antibody (BioXCell, Lebanon, NH, USA) was freshly diluted to 2.5 mg/mL in the diluted buffer. Mice were i.p. injected with αPD-L1 as 10 mg/kg mice body weight for five consecutive days. Mice were sacrificed at day five post-RT, and tumor weights were measured. For the survival assay, the mouse’s death was recorded when signs were shown (lethargy, failure to ambulate, and loss of more than 20% of original body weight). To measure the immune response, the splenocytes were harvested for the ex-vivo cytotoxic assay by the CytoTox 96^®^ Non-Radioactive Cytotoxicity Assay (Promega, Madison, WI, USA) according to the manufacturer’s procedures.

### 4.5. Immunohistochemical Analysis 

To label the tumor hypoxia region, mice were i.p injected with pimonidazole (PIMO) hydrochloride (4 mg per mouse, Hypoxyprobe^TM^-1 Kit, Hypoxyprobe, Inc, Burlington, MA, USA) one hour before sacrifice. To mimic small molecule drug penetration, mice were injected with 100 μL of Hoechst33342 (10 mg/mL, Thermo Fisher, Waltham, MA, USA) from orbital sinus 10 mins before sacrifice. The tumor tissues were embedded with the OCT compound (Sakura Finetek, Torrance, CA, USA) and stored at the −80 °C.

The section slides were fixed with methanol and permeabilized with 0.05% Tween-20 (Sigma, St. Louis, MO, USA). The slides were subsequently mounted with blocking buffer (4% FBS and 1% goat serum in PBS) for one hour to prevent non-specific binding. After blocking, the first antibodies were used as follows: CD68, F4/80 (Bio-Rad, Hercules, CA, USA), CD11b, CD31, CD4, CD8 (BD Pharmingen, San Jose, CA, USA), NG-2 (Millipore, Burlington, MA, USA), PD-L1, and PD-1(R&D, Minneapolis, MN, USA). The slides were stained overnight in 4 °C and then washed. The specific host secondary antibodies conjugated with Alexa Fluor 488 (Invitrogen, Waltham, MA, USA), Alexa Fluor 594 (Invitrogen), and Cy5 (Invitrogen) were mounted at room temperature for one hour. For in vivo αPD-L1 drug (BioXCell) localization, the slides were blocked and stained with secondary antibody. Slides were mounted with DAPI (Invitrogen) for the nucleus visualization. Images were taken by the AxioCam MRC-5 camera on an Axiovertskop 40 fluorescence microscope (Carl Zeiss, Jena, Germany) and analyzed by Image-Pro Plus 6.0 (Media Cybernetics, Inc., Rockville, MD, USA) and ImageJ 1.48v software. Quantification was calculated as the proportion of the whole tumor region (positive pixels/tumor area pixels × 100%).

### 4.6. Flow Cytometry Analysis

Blood were collected and mixed with RBC lysis buffer (eBioscience, Carlsbad, CA, USA) to lyse red blood cells for 5 min. The same volume of PBS was applied to stop the RBC lysis reaction, and the cell pellet was resuspended in PBS. Cell suspensions were blocked with 1% goat serum (Gibco) and 0.2% Fc blocking reagent (BD Pharmingen) for 30 min. After blocking, cells were stained with fluorescence conjugated antibodies against CD11b, Ly6C, Ly6G, or CD45 (BD Phamingen) for 30 minutes on ice. The cell suspensions were washed twice by PBS before analysis on a BD FACSCanto^TM^ flow cytometer (Becton Dickinson, Franklin Lakes, NJ, USA) and data were analyzed by FACSDiva software v6.1.3 (Becton Dickinson).

### 4.7. Statistics

Statistics were performed using the two-tailed Student’s t-test or the one-way ANOVA by Prism 5.0 (GraphPad, San Diego, CA, USA). A *p* value ≤ 0.05 was regarded as statistically significant.

## Data Availability

The data presented in this study are available within the article text, figures, and Supplementary Materials.

## Supplementary Materials

The following are available online at https://www.mdpi.com/1422-0067/22/4/2091/s1, Figure S1: The change of tumor-infiltrating CD4+ cells after various RT protocols, Figure S2: PD-1 expression was upregulated in CD8+ T cells after SHD-RT, Figure S3: The coverage of pericytes on vessels was decreased after SHD-RT.
