# Supplementary material for: Ablative Radiotherapy Reprograms the Tumor Microenvironment of a Pancreatic Tumor in Favoring the Immune Checkpoint Blockade Therapy
Source: Int J Mol Sci. 2021 Feb 19;22(4):2091. doi: 10.3390/ijms22042091 (PMC7923299; doi:10.3390/ijms22042091)
Supplement: Supplementary file 1 [file ijms-22-02091-s001.zip › Supplementary Material/Supplementary Material.docx]

**Supplementary Materials**

**Figure S1. The change of tumor-infiltrating CD4+ cells after various RT protocols.** The representative images of CD4+ staining on tumor tissues from different experimental groups. Each dot represents one mouse data, and each mouse data counted at least five tumor sections.

**Figure S2. PD-1 expression was upregulated in CD8+ T cells after SHD-RT.** (A) Representative images of PD-1 (green) and CD8 (red) staining. (B) The percentage of PD-1+ CD8+ cells was quantified. ***: *P*< 0.001. (C) The experimental scheme for the SHD-RT and the procedure of anti-CD8 antibody administration. (D) The number of CD8+ cells in the blood and tumors after treatment was analyzed by flow cytometry. *: *P*< 0.05.

**Figure S3. The coverage of pericytes on vessels was decreased after SHD-RT.** (A) Representative images of NG2 (green) and CD31 (red) staining. (B) The coverage of pericytes on vessels was quantified. **: *P*< 0.01.
