# Supplementary figures and images for: Ablative Radiotherapy Reprograms the Tumor Microenvironment of a Pancreatic Tumor in Favoring the Immune Checkpoint Blockade Therapy
Source: Int J Mol Sci. 2021 Feb 19;22(4):2091. doi: 10.3390/ijms22042091 (PMC7923299; doi:10.3390/ijms22042091)

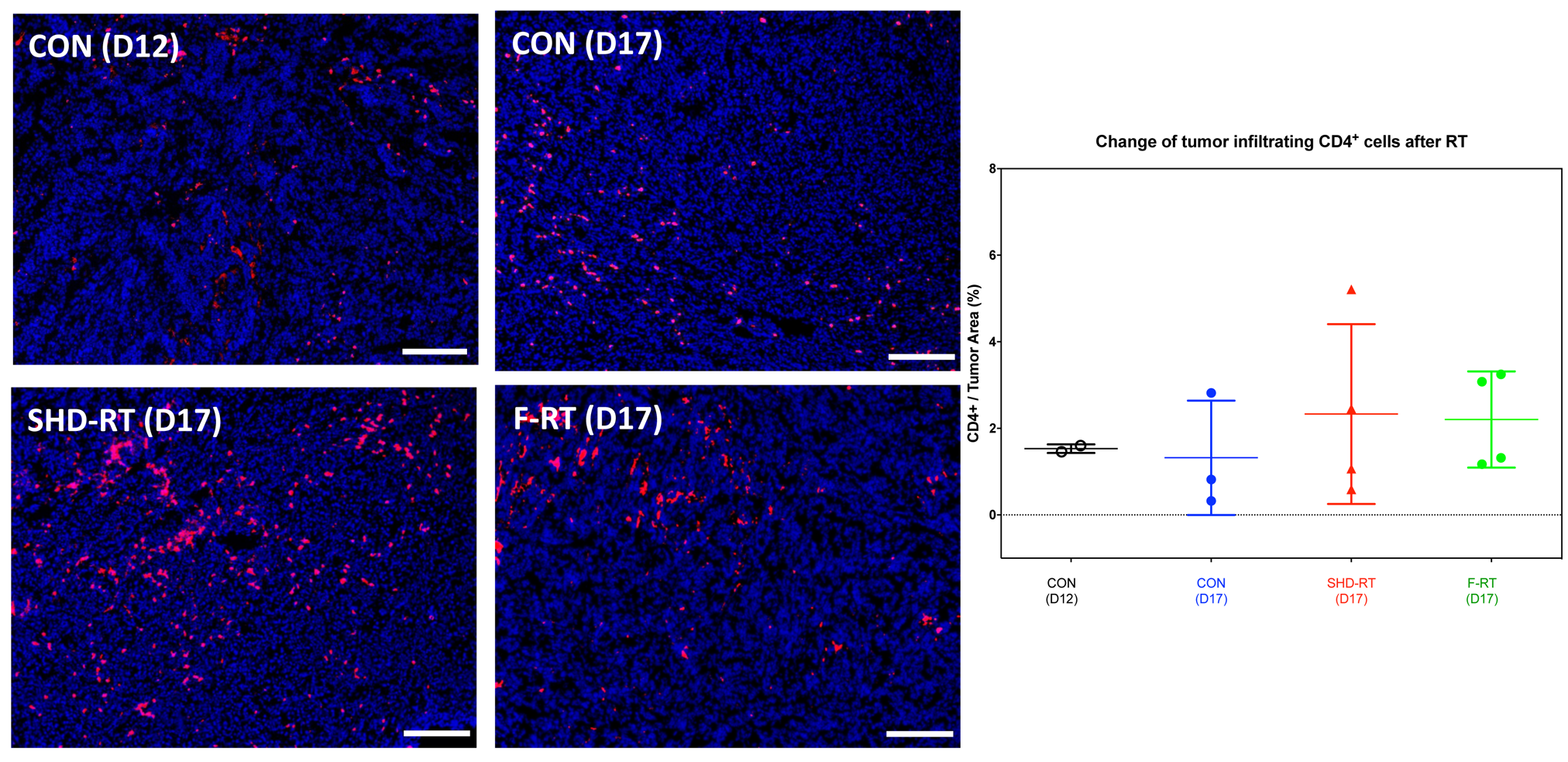

Supplement: Supplementary file 1 [file ijms-22-02091-s001.zip › Supplementary Material/Figure S1.tif]

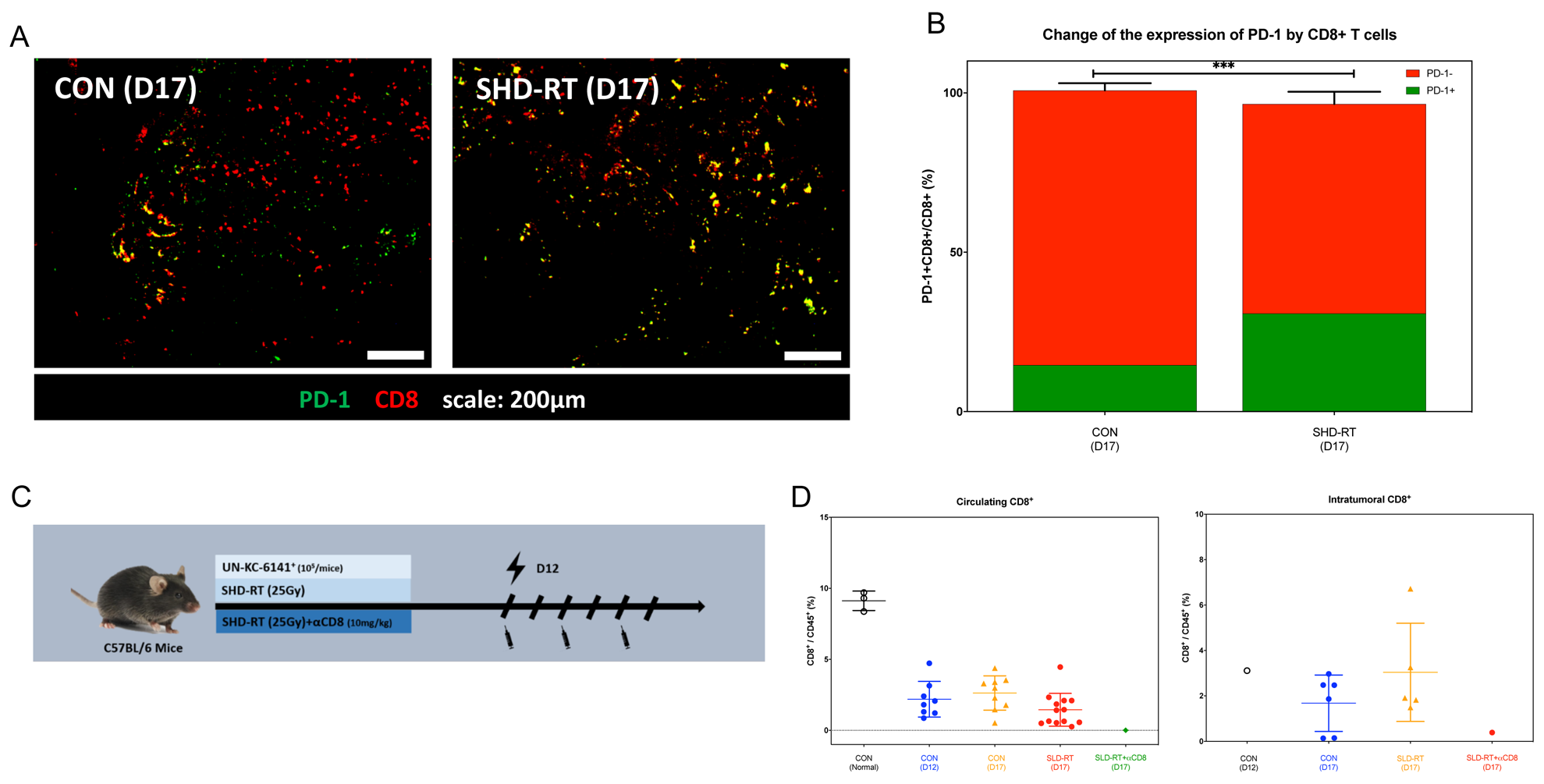

Supplement: Supplementary file 1 [file ijms-22-02091-s001.zip › Supplementary Material/Figure S2.tif]

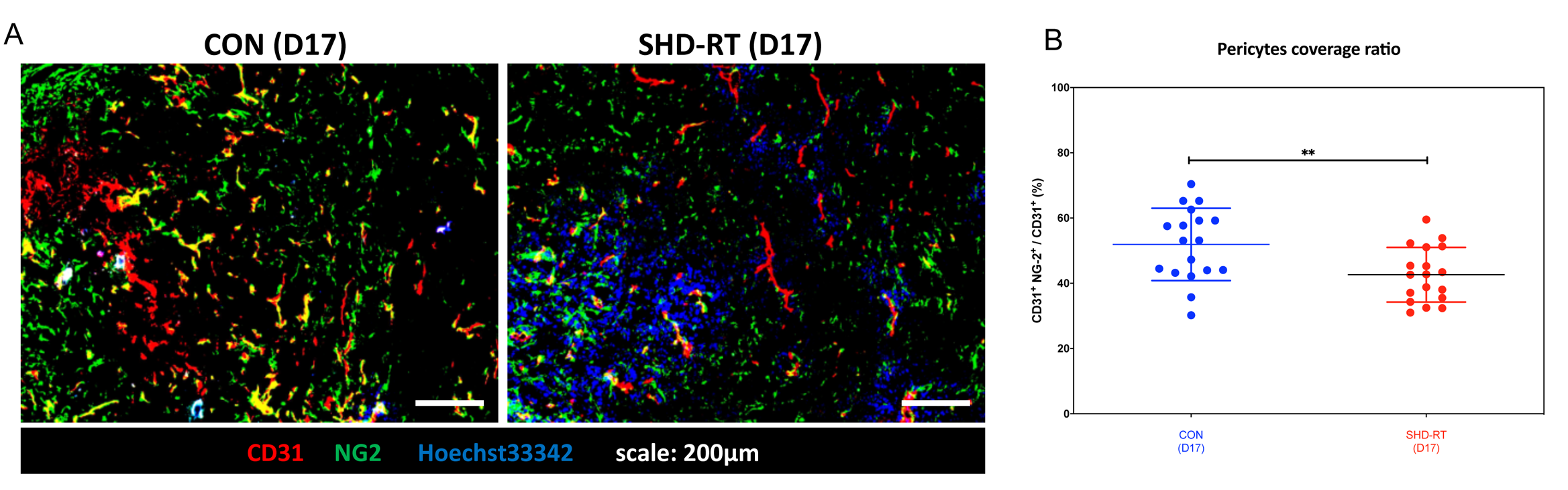

Supplement: Supplementary file 1 [file ijms-22-02091-s001.zip › Supplementary Material/Figure S3.tif]
